# The epidemiology of bacterial zoonoses in pastoral and dairy cattle in Cameroon, Central Africa

**DOI:** 10.1111/zph.12865

**Published:** 2021-06-15

**Authors:** Robert F. Kelly, Amy Jennings, Jennifer Hunt, Saidou M. Hamman, Stella Mazeri, Egbe F. Nkongho, Victor N. Ngwa, Vincent Tanya, Melissa Sander, Lucy Ndip, Paul R. Bessell, Kenton L. Morgan, Ian G. Handel, Adrian Muwonge, Barend M. de C Bronsvoort

**Affiliations:** 1 Royal (Dick) School of Veterinary Studies and The Roslin Institute, University of Edinburgh, Easter Bush, Midlothian, UK; 2 Regional Centre of Wakwa, Institute of Agricultural Research for Development, Ngaoundere, Cameroon; 3 School of Life Sciences, University of Lincoln, Lincoln, UK; 4 School of Veterinary Sciences, University of Ngaoundere, Ngaoundere, Cameroon; 5 Cameroon Academy of Sciences, Yaoundé, Cameroon; 6 Tuberculosis Reference Laboratory Bamenda, Hospital Roundabout, Bamenda, Cameroon; 7 Laboratory of Emerging Infectious Diseases, University of Buea, Buea, Cameroon; 8 Institute of Ageing and Chronic Disease and School of Veterinary Science, University of Liverpool, Neston, UK

**Keywords:** brucellosis, cattle, epidemiology, leptospirosis, Q fever, zoonoses

## Abstract

Previous work identified that bacterial zoonoses (*Brucella species, Coxiella burnetii* and *Leptospira hardjo)* were present in Cameroonian pastoral cattle. To assess the characteristics of this zoonotic risk, we analyse seroprevalence of each pathogen and the associated management, herd and environmental factors in Cameroonian pastoral and dairy cattle. Cross-sectional samples included pastoralist herds in the Northwest Region (NWR *n* = 750) and Vina Division (VD *n* = 748) and small holder dairy herds in the NWR (*n* = 60). Exposure to *Brucella* spp., *C. burnetii* and *L. hardjo* were screened for using commercial ELISAs and population adjusted estimates made. In addition, individual, herd and ecological metadata were collected and used to identify risk factors associated with animal-level seropositivity. In the pastoral cattle, seroprevalence to *Brucella spp*. was relatively low but was higher in the NWR (4.2%, CI: 2.5%–7.0%) than the VD (1.1%: CI 0.5%–2.4%), while *L. hardjo* seroprevalence was much higher though similar in the NWR (30.7%, CI 26.3%–35.5%) and VD (35.9%, CI 31.3%–40.7%). No differences were noted in *C. burnetii* seroprevalence between the two study sites (NWR: 14.6%, CI 11.8%–18.0%. VD: 12.4%, 9.6%–15.9%). Compared to pastoral, dairy cattle had lower seroprevalences for *L. hardjo* (1.7%, CI: 0.0%–4.9%), *C. burnetii* (0.0%, CI 0.0%–6.0%) but similar for *Brucella* spp. (5.0%, CI 0.0%–10.6%). Increased odds of *Brucella spp*. seropositivity were associated with owning sheep or rearing sheep and fencing cattle in at night. Adult cattle had increased odds of being seropositive for both *C. burnetii* and *L. hardjo*. Additionally, exposure to *C. burnetii* was associated with local ecological conditions and *L. hardjo* was negatively associated with cattle undertaking transhumance. This work highlights that exposure to these 3 important production diseases and occupational zoonoses are widespread in Cameroonian cattle. Further work is required to understand transmission dynamics between humans and livestock to inform implementation of effective control measures.

## 1 Introduction

Cattle rearing is nutritionally, economically and culturally important to rural livelihoods in sub-Saharan Africa (SSA) (Dessie & Okeyo Mwai, 2019), with livestock contributing ~30% of the continent’s agricultural gross domestic product (FAO, 2020). Over the last decade, rapid population growth and urbanization in SSA has led to increased demand for meat and dairy products (Thornton, 2010). Consequently, intensification of cattle production systems is seen as an important part of economic development (Otte et al., 2019). Despite their value, cattle also pose a risk to human populations by acting as a source of zoonotic infections. Cattle zoonoses can be transmitted either through direct close contact or indirectly through the food chain to consumers. The burden of food borne zoonoses is likely significant in SSA countries (Gebreyes et al., 2014), due to limited awareness of the risks posed from consuming animal products (Tebug et al., 2015) and limited implementation of food safety practices (El Zowalaty et al., 2019). Human infections are often associated with individuals with greatest animal contacts such as animal keepers, abattoir workers and those that consume unpasteurized milk (Dadar et al., 2020; Mgode et al., 2015; Pereira et al., 2020; Vanderburg et al., 2014).

Bacterial zoonoses such as *Brucella abortus* (the major *Brucella* spp. in bovine populations), *Coxiella burnetii* (Q fever) and *Leptospira interrogans serovar hardjo* (the major *L. hardjo*) have been reported in cattle and human populations in SSA (Ducrotoy et al., 2017; Mgode et al., 2015; Vanderburg et al., 2014). In low-middle income countries (LMICs), bacterial zoonoses account for 3.4% of severe febrile illnesses that have been assumed to be malaria (Prasad et al., 2015). Bacterial zoonoses also have an impact on cattle productivity through losses from infertility and abortions and therefore can negatively impact cattle-related livelihoods. In cattle, *B. abortus, C. burnetii* and *L. hardjo* can be associated with abortion, weak calves and milk drop (Colville & Berryhill, 2007; Neta et al., 2010; Scott et al., 2011) as well as a number of specific clinical signs associated with each infection (Colville & Berryhill, 2007; Neta et al., 2010). In both animals and humans, acute and chronic clinical presentations are possible for these three infections. For example, *C. burnetii* and *L. hardjo* can be associated with persistent infertility and repeated abortion. Persistent herd infections can lead to significant production losses regardless of the production system, including in smallholder communities where few cattle are kept (Salmon et al., 2018) and pastoral herds where 10 s–100 s of cattle are kept as the predominant form of financial capital (Sheik-Mohamed & Velema, 1999). Latent infections also pose a hidden health risk to both cattle and human populations, due to the asymptomatic nature of infections and limited routine diagnostic surveillance. Diagnoses of zoonoses in humans and animals are challenging because of these asymptomatic and indistinct clinical presentations. Misdiagnosis can easily occur without access to advanced diagnostic technologies (Maze et al., 2018) and consequently lead to poor treatment outcomes (El Zowalaty et al., 2019).

Despite the potential risk of zoonosis transmission from cattle to humans, understanding of transmission networks is patchy and poorly monitored in SSA (Gebreyes et al., 2014), with medical and veterinary infrastructure often fragmented and under-resourced. To mitigate the risks posed by bacterial zoonoses shared between cattle and humans, it is critical to understand the epidemiology of pathogens within local ecosystems and identify risk factors that drive transmission. For example, the Central African country of Cameroon has a cattle population of ~6 million kept mainly on the fertile mountainous west and northern savannah regions of the country (United Nations Statistics Division, 2011). The majority of herds are reared in pastoral grazing systems in herds of 10–100 s of cattle by the Fulani people, who graze and trade cattle across the wider Central-West African region (Motta et al., 2017). Cattle rearing is central to Fulani community life, with cattle kept as a source of wealth, local milk production and meat for distant urban populations (Frantz, 1993; Kelly et al., 2016). An increasing demand for meat and milk in urban populations over the past 40 years (Bayemi et al., 2005) has encouraged growth of pastoral cattle populations across the country and the emergence of peri-urban dairy smallholders in the North West Region as part of a local economic development programme in the early 1990s. Dairy smallholders keep dairy cattle in housed ‘cut and carry’ feeding systems in herds of usually under 10 cattle (Kameni et al., 1999; Njew et al., 2002). A previous study in the early 2000s highlighted the presence of bacterial zoonoses in pastoral cattle populations in the Adamawa Region of Cameroon (Mazeri et al., 2012; Scolamacchia et al., 2010). As cattle are closely linked to community livelihoods, further understanding of bacterial zoonosis epidemiology may encourage investment in collaborative public and animal health campaigns (Sheik-Mohamed & Velema, 1999).

In this paper, we leverage an integrated data resource generated during the largest bovine tuberculosis survey in Cameroon. We use a serum bank generated from the population-based sample of pastoral and small holder dairy herds to screen for antibodies to *Brucella spp*., *C. burnetiii* and *Leptospira interrogans serovar hardjo*. Along with metadata collected, we estimate the seroprevalence and identify the associated risk factors for exposure to each infection in these cattle populations in Cameroon.

## 2 Materials And Methods

### 2.1 Study design

**Figure 1:**
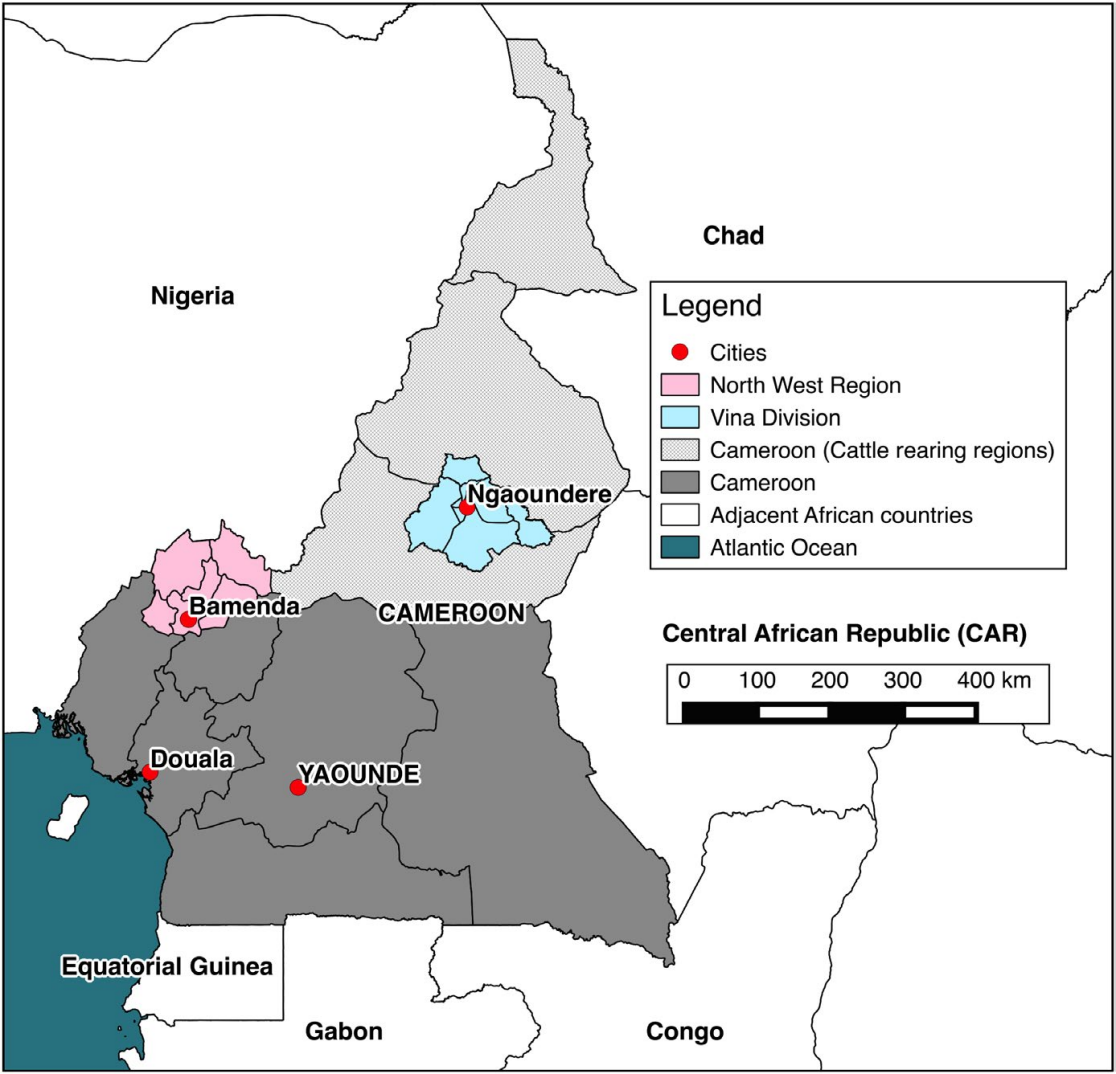
Map of Cameroon. The location of cattle rearing areas (light grey), study sites (pink and blue) and major cities (red)

This project was part of a larger project focussed on increasing understanding of bovine tuberculosis (bTB) and liver fluke in Cameroonian cattle populations, with the original justification of study designs described elsewhere (Kelly et al., 2016). We utilize bovine serum samples, animal and herd data from this project to investigate the seroprevalence of bacterial zoonoses and associated risk factors in Cameroonian cattle populations. The study sites were the North West Region (NWR) and Vina Division (VD) of the Adamawa Region of Cameroon (Figure 1). Cross-sectional studies sampled two pastoralist populations (NWR & VD) and three cooperatives of small-scale dairy farmers (NWR only). The Ministry of Livestock, Fisheries and Industrial Agriculture/Ministere de l’Elevage des Peches et Industries Animales (MINEPIA) provides veterinary services through local veterinary technicians stationed at Zootechnical and Veterinary Sanitary Control Centres (ZVSCC) distributed all across the country (Kelly, 2017). MINEPIA cattle population records were the basis for sample frames. Sampling was conducted between January–May 2013 in the NWR and September-November 2013 in the VD, respectively.

Pastoral cattle populations in the NWR and VD were estimated to be 506,548 and 176,257, respectively, from vaccination records collected by MINEPIA (Department & C. N. S, 2012). The pastoral cattle population eligible for sampling were herds listed in vaccination records at 81 ZVSCCs in the NWR and 31 ZVSCCs in the VD in 2012. There were 5,053 cattle herds in the NWR and 1,927 in the VD, with a range of 1–215 animals per herd. A population weighted stratified random sample of registered herds was sampled in each of the two study sites. The sample was stratified by sub-location within each administrative area, as seven Divisions in the NWR and eight sub-Divisions in the VD. The number of herds sampled from each sub-location was proportional to the total number of herds within that sub-location. The sample size for this bTB focussed project was based on a clustered random sample with an estimated animal-level bTB prevalence of ~10%, a within herd variance of 0.15 and between herd variance of 0.01, an average herd size of 70, a relative cost of 12:1 for herd:animal and relative error of ±15% (Survey Toolbox; AusVet) (Sergeant, 2015). This gave 1,399 pastoralist cattle; rounded to 1,500 for ease of selection, with a target sample size of 15 cattle per herd and 88 herds under the simplifying assumption of perfect test performance. A final sample was of 100 herds with 50 at each site. Within each herd, the 15 samples were stratified to each of three age classes: young (<2 years: dentition score (DS) 0), adult (≥2 and <5 years: DS 1–4) and old (≥5 years: DS 5). If there were insufficient animals of one age group, additional animals of any age were sampled.

The sampling frame for the dairy cattle population was based on the 2012 registration lists of small-scale dairy farmers held by MINEPIA, with addresses obtained from the NWR office in Bamenda (Kelly, 2017). In total, 164 farmers and 492 cattle from the three main cooperatives were included in the sampling frame. For simplicity, a random sample of cattle was selected based on the assumptions of perfect tests and a bTB prevalence of 6% (Egbe et al., 2016). As the majority of farmers only had one or two adult cattle, 46 farmers were randomly selected proportional to the number of farmers in each cooperative.

### 2.2 Data collection

Pastoral herds were visited either at a site where the cattle grazed or were handled. Dairy herds were visited at the homestead. A local translator explained the project to the pastoralist herdsman or dairy farmer in either Foulfulde, Pidgin English or French. Individuals were asked to give verbal consent to participating in the study in the language in which they were most comfortable.

All data were initially recorded onto paper forms, which were later transferred into a relational Access database (Microsoft Access^®^). From each herd, 15 animals were selected by the same local translator. The local translator was unaware of individual animal’s health status and selected animals nearest to sample. The total number of cattle present and location using GPS coordinates (using Garmin eTrex^®^ Venture) was recorded. All sampled animals were then examined by the same veterinarian, with data recorded at an individual animal level to accompany samples. Animal data collection included recording animal signalment (sex, age by dentition, breed, body condition score [BCS]) if anthelmintic treatment had been administered in the previous 12 months. The method for ageing by dentition and BCS was carried out on 5-point scales (Egbe et al., 2016). Cattle breeds were based on phenotypic appearance, for *Bos indicus* (Fulani and Gudali) and *Bos taurus* (Holstein Friesian) breeds. ‘Mixed breed’ cattle were defined as cattle that had the phenotypic appearance of mixed *Bos indicus* breeding. Plain and heparinized blood samples were collected from the jugular or tail vein prior processing. A herd-level questionnaire was administered by interview in the respondents preferred language. The questionnaire collected herd-level data on husbandry and dairying practices, knowledge and awareness of infectious diseases (Appendix S1). The herd-level descriptive results have been published previously (Kelly et al., 2016).

### 2.3 Diagnostic tests for zoonoses in cattle

The laboratory work described in this paper was conducted after the bTB project was completed. After collection, all serum samples were heat treated at 56°C for 120 min and stored at −20°C until tested. Screening for *Brucella spp*. (*B. abortus, B. melitensis* and *B. suis*) exposure was conducted using the ID Screen^®^ Brucellosis Serum Indirect Multi-species ELISA (ID.Vet, n.d.-a). In brief, 11 μl of each serum sample was diluted to 1:20 with the provided dilution buffer. The diluted sample was added to a well on the purified *Brucella* LPS coated microplate and incubated at 21°C for 45 min. The microplate was washed three times with 300 μl of wash solution, and 100 μl of supplied conjugate was added for 30 min at 21°C. Using the same method, the microplate was washed again and 100 μl of substrate solution was added to each well for 15 min at 21°C. To stop the reaction, 100 μl of stop solution was added to each well and the microplate was read at 450 nm. For a microplate to be considered valid, the mean of the duplicate positive (PC) and negative (NC) controls were calculated. For a microplate to pass, mean PC optical density (OD) needed to be >0.35 and the mean positive and negative OD ratio (ODPC/ODNC) needed to be >3. For each sample, the sample to positive ratio (S/P%) was calculated by (ODsample – ODNC)/(ODPC –ODNC) × 100. The manufacturers suggest samples S/P% ≤ 110% are considered negative; S/P% > 110% and <120% are considered doubtful, and S/P% ≥ 120% are considered positive. For this study, samples S/P% < 120% are considered negative and S/P% ≥ 120% are considered positive.

Screening for *C. burnetii* exposure was conducted using the ID Screen^®^ Q Fever Indirect Multi-species ELISA (ID.Vet, n.d.-b). Briefly, 15 μl of each serum sample was diluted to 1:10 with the provided dilution buffer. The diluted sample was added to a well on the *C. burnetii* phase I and II strain coated microplate. The ELISA, validation and S/P% methods were the same as described for *Brucella* spp. ELISA. The manufacturers suggest samples S/P% ≤ 40% are considered negative S/P% 40% and ≤50% are considered doubtful and S/P% ≥ 50% are considered positive. For this study, samples S/P% ≤ 50% are considered negative and S/P% > 50% are considered positive.

Screening for *L. hardjo* exposure was conducted using the PrioCHECK^®^ *L. hardjo* Indirect ELISA (Thermo Fisher, n.d.). In brief, 10 μl of each serum sample was diluted to 1:200 with the provided dilution buffer. The diluted sample was added to a well on the inactivated *Leptospira interrogans serovar hardjo* antigen coated microplate and incubated at 37°C for 60 min. The microplate was washed six times with 300 μl of wash solution, and 100 μl of supplied conjugate was added for 60 min at 37°C. Using the same method, the microplate was washed again and 100 μl of chromogen substrate solution was added to each well for 15 min at 22°C. To stop the reaction, 100 μl of stop solution was added to each well and the microplate was read at 450 nm. For diagnostic interpretation, per cent positivity (PP) was calculated for each reference and sample well (OD test well/OD reference sample 1 × 100). For a microplate to be considered valid, duplicated reference wells needed to meet specific criteria including: blank well mean OD <0.150, reference serum 1 mean OD ≥1.0, reference serum 2 mean PP <20, reference serum 3 mean PP ≥20 and <60. The manufacturers suggest samples PP <20% are considered negative, PP ≥20% and ≤45% are considered inconclusive and PP >45% are considered positive. For this study, samples PP <20% are considered negative and PP ≥20% are considered positive.

### 2.4 Statistical analysis

All statistical analyses were performed using packages and functions in R version 3.6.1 (RStudio Team, 2020). Graphics were produced using the *ggplot2* package (Wickham, 2009). Spatial data were displayed using QGIS 2.2^®^ (Team & Q. D., 2009) or *tidyverse* collection of R packages (Hadley Wickham, 2017) and shape files obtained from the GADM database of Global Administrative Areas (www.gadm.org). The structure of the pastoralist survey was incorporated into analyses using the *svydesign, confint* and *svyby* functions in the *survey* package (Lumley, 2004). This allowed the stratified study designs to be accounted for in descriptive statistics by taking into account proportional sampling by number of herds in each study site sub-location (NWR by division and VD by subdivision) and accounting for herd clustering of sampled animals. For reporting, cattle BCS variable was collapsed to thin (1–2), moderate (3) and fat (4–5) categories. Dentition score was collapsed to young (<2 years: DS 0), adult (≥2 and <5 years: DS 1–4) and old adult (≥5 years: DS 5). Confidence intervals (CI) are reported at 95% level throughout. Specific differences in sample statistics were identified by non-overlapping CIs (Schenker & Gentleman, 2001).

Mixed effect multivariable logistic regression (MLR) models were used to investigate dichotomous test outcome (seropositive or seronegative) for exposure to each pathogen at the individual animal level. Logistic regression models were developed using the R package *stats* (R Core Team, 2019) and *lme4* (Bates et al., 2015). Univariable analysis was used to screen the biologically plausible explanatory variables (Kleinbaum & Klein, 2010). Potential risk factors at individual, herd and ecological level were investigated as explanatory variables. Ecological variables from three sources were recentred around zero and normalized their distribution prior analysis, using log transformation: log(*x*)−min(log(*x*)) (Appendix S2). Explanatory variables were included in final MLR model selection if their *p* value <0.2 (Kleinbaum & Klein, 2010). Potential biological correlations were assessed by calculating the phi coefficient using the *psych* package (Revelle, 2020). If phi was ≥0.5, two variables were considered correlated and the explanatory variable with the highest p value was selected. To account for the study design, herd was included as a random effect and study site as a fixed effect in all multivariable models. Backwards stepwise selection using the Akaike information criterion (AIC) (Dohoo et al., 2009) was used for parsimonious model selection (Appendix S3). As part of this process, interactions between variables included as fixed effects were investigated throughout the model building process including age, sex and study site.

### 2.5 Ethical statement

The study was reviewed and approved by the University of Edinburgh Ethics Committee, UK (ERC No: OS02-13), and by the Institute of Research and Development (IRAD), Cameroon. All participants gave informed verbal consent to the translator before participating and could opt out at any stage.

## 3 Results

### 3.1 Cattle sample

In total, 100 pastoralist herds were recruited: 50 in the NWR and 50 in the VD. Of these 100, 23 were replacements from the same ZVSCC for herds that declined or were unable to participate in the study. All 46 selected dairy farmers participated, and none were replaced. In total, 750 pastoral cattle were sampled from 50 herds (15 per herd) in the NWR and 748 pastoral cattle from 50 herds (14–15 per herd) in the VD in the pastoral cattle study. In the dairy cross-sectional study, 60 cattle (1–4 per herd) were sampled from 46 dairy farmers. Herd-level data used for risk factor analysis have been published previously (Kelly et al., 2016) and are described in detail in Appendix S1.

**Figure 2:**
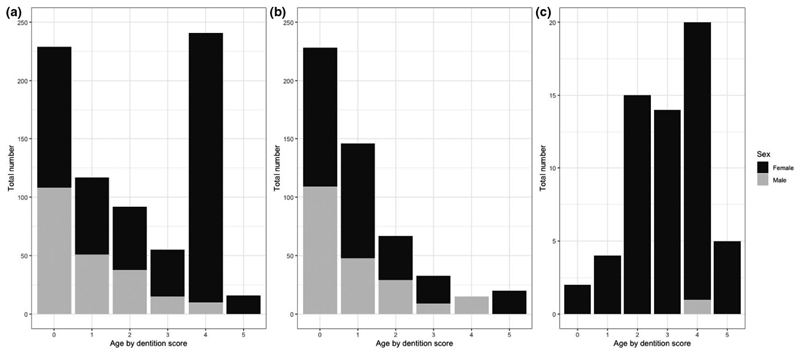
Proportions of cattle sample by dentition score, sex and by study site grouping. (a) NWR pastoral cattle (*n* = 750), (b) VN pastoral cattle (*n* = 748), (c) NWR dairy cattle (*n* = 60)

For pastoral cattle, there were equal numbers of male and females in the young age group (DS 0) and mainly females in the adult groups (Table 1). As the dairy herds were all very small, all animals in a herd were sampled, so there was no age structure to the sampling. Consequently, the sample represented the age of the dairy population and these were mainly adult female animals (Table 1 and Figure 2). Pastoral cattle in the NWR had lower BCSs than both those in the VD and the dairy cattle. The majority of dairy animals had been treated with an anthelmintic in the previous 12 months, compared to less than half those in pastoral herds (Table 1).

### 3.2 Seroprevalence of zoonotic infections

**Figure 3:**
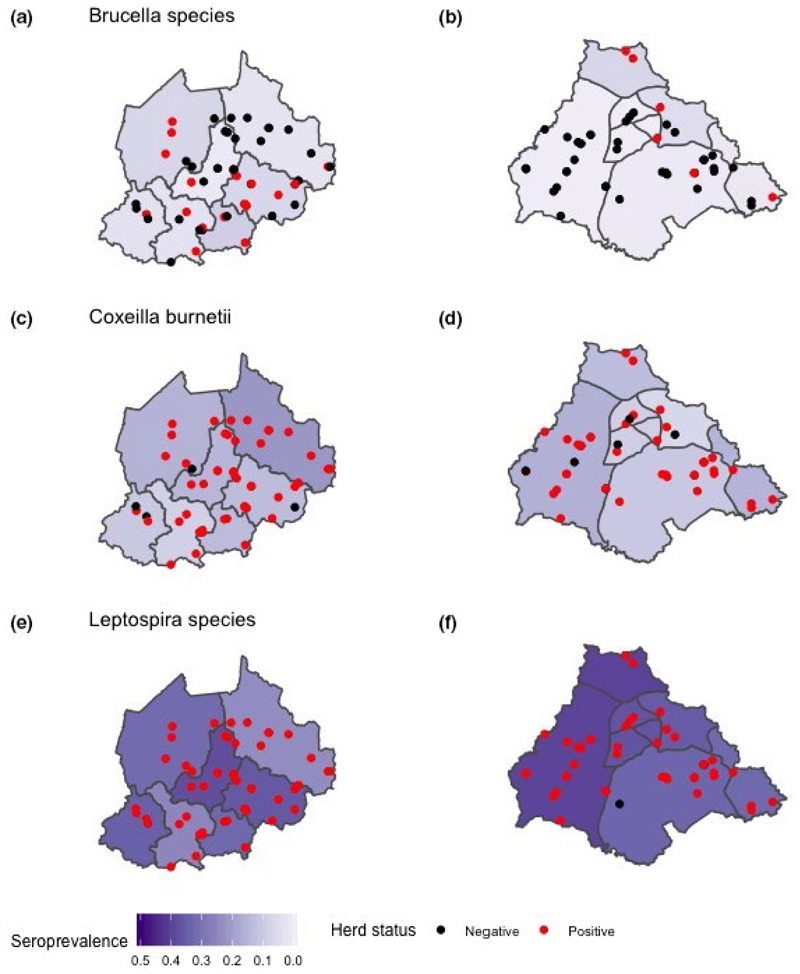
Map study site by sub-location prevalence for pastoral cattle (subfigure a, c, e: NWR *n* = 750. b, d, f: VD *n* = 748). a–b: *Brucella* spp., c–d: *Coxeilla burnetii* and e–f: *Leptospira interrogans serovar hardjo*. Positive herds have at least one infected animal per herd

**Figure 4:**
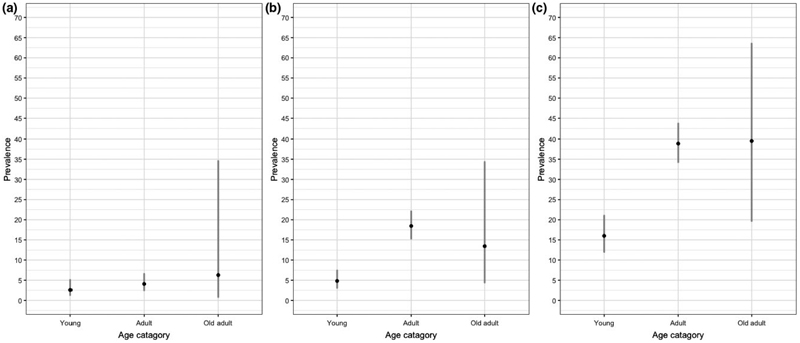
Relationship between prevalence and age in pastoral cattle (*n* = 1,498). (a) *Brucella* spp., (b) *Coxeilla burnetii* and (c) *L. hardjo*

**Figure 5:**
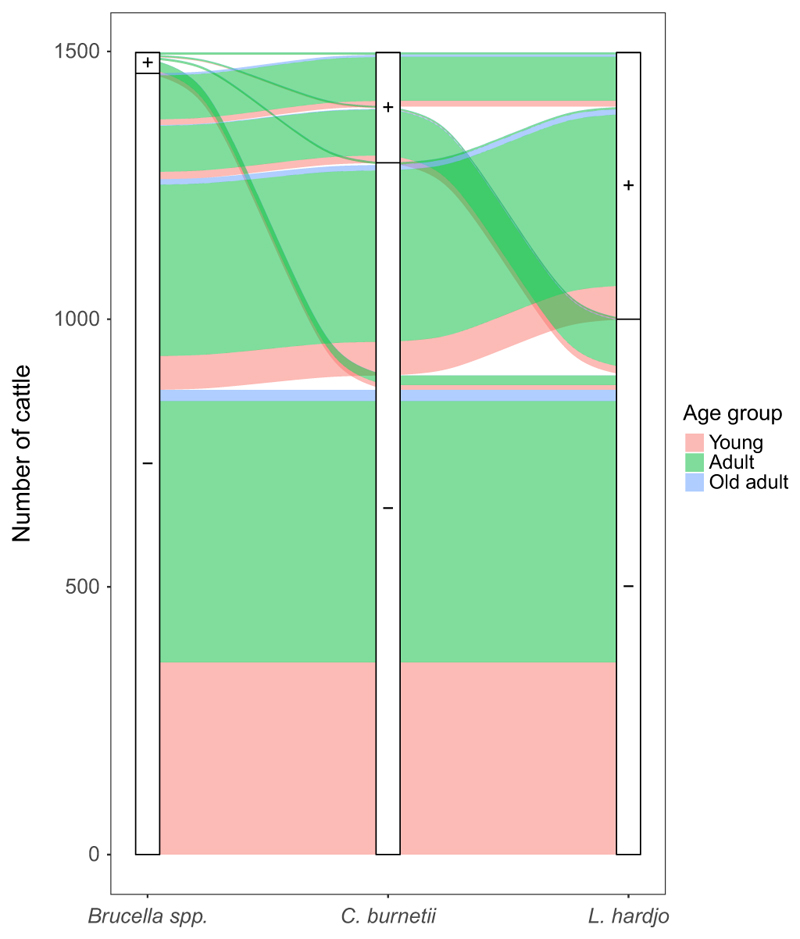
Ribbon plot to represent the relationship between zoonoses co-exposure and age in pastoral cattle (*n* = 1,498). Each white bar represents the number of animals positive or negative for each zoonosis. Coloured ribbons represent the number of animals by age group by zoonosis. The width of each coloured ribbon connecting two bars represents the number of animals by co-infection combination

For pastoral cattle, *Brucella* spp. animal-level seroprevalence was higher in NWR compared to VD. No differences were noted in *C. burnetii* seroprevalence between the two study sites. *L. hardjo* seroprevalence was considerably higher than either *Brucella* spp. or *C. burnetii* seroprevalences in the NWR and VD (Table 2). The adjusted animal-level seroprevalences by administrative area are given in Figure 3 with positive herds highlighted (at least 1 positive animal per herd). When investigated by age group, *C. burnetii* and *L. hardjo* seroprevalences were higher in adult compared to young cattle (Figure 4). A number of pastoral cattle were co-exposed to each zoonoses (Figure 5), with adult age groups more frequently exposed to *C. burnetii* and *L. hardjo*.

For dairy cattle, there were no differences in seroprevalence for the 3 bacterial zoonoses. Compared to pastoral cattle, *Brucella spp*. seroprevalence was similar to pastoral cattle but much lower for *C. burnetii* and *L. hardjo*. Seropositivity in dairy cattle was not associated with age as the majority were adults (Table 1).

### 3.3 Risk factors for zoonotic infections

For each zoonosis, following an initial univariable screening (Appendix S2), a multivariable mixed effects model was developed to identify and quantify animal, husbandry and ecological factors associated with seropositivity of pastoral cattle (Appendix S3). The final model indicated that owning or rearing sheep and fencing cattle at night were all associated with an increased odds of being *Brucella* spp. seropositive (Table 3). Being positive for *C. burnetii* was significantly associated with an increased odds of being positive for *L. hardjo* and vice versa (Tables 4 And 5). In other words, animals were more likely than chance to be seropositive for both these infections than to one or the other. Adult cattle (those over two years old) were at increased odds of being seropositive to both *C. burnetii* and *L. hardjo*. For *C. burnetii*, decreased precipitation and increased tree density increased the odds of seropositivity. For *L. hardjo*, undertaking transhumance was protective and decreased the odds of seropositivity. For each final model, the proportion of the variance explained by herd-level sampling structure (random effects) was assessed by calculating the interclass correlation coefficient (Appendix S3). The proportion of variance accounted for was small (ranged between 0.021 and 0.137), and removal did not improve model performance.

For dairy cattle, risk factors were not investigated given the low seroprevalence and the little variation in husbandry practices between herds.

## 4 Discussion

We highlighted that *Brucella spp*., *C. burnetii* and *L. hardjo* continue to circulate in pastoral herds and were able to identify risk factors associated with cattle seropositivity in Cameroon. We also detected exposure to *Brucella spp*. and *L. hardjo* in dairy cattle. Although we used historically collected samples, this is the most recent comprehensive surveillance of cattle exposure to bacterial zoonoses in Cameroon. This study highlights the benefit of long-term storage of biological samples collected from field studies for infectious disease surveillance in livestock in resource-limited settings.

Previous estimates of *Brucella* spp. seroprevalence in pastoral cattle in Cameroon (estimated individual animal prevalence in the NWR (4.4%) (Kong et al., 2016), Adamawa (3.1%–11.5%) (Awah-Ndukum, Mouiche, Bayang, et al., 2018; Scolamacchia et al., 2010) and North Regions (6.1%) (Awah-Ndukum, Mouiche, Bayang, et al., 2018)) and other Central-West African countries (Chad (7%) (Schelling et al., 2003), Niger (0.5%–4.6%) (Razac Boukary et al., 2013) and Togo (7.3%–9.2%) (Dean et al., 2013)) were similar to our study estimates. Although one study from Nigeria showed higher estimates of 15.9%–45.1% (Ducrotoy et al., 2014), highlighting the potential increased local significance. Close contact with bovines has been identified in pastoral systems elsewhere in SSA as a risk factor for exposure to *Brucella spp*. For example, association is reported with increased herd size, increased contact between herds, shared grazing and contact with buffalos (McDermott & Arimi, 2002). In our study, fencing pastoral cattle at night was identified as a risk factor for exposure to *Brucella spp*., potentially due to increased close contact with infected cattle. Keeping sheep was also associated with *Brucella spp*. seropositivity. *Brucella spp*. infections are commonly attributed to *B. abortus* and *B. melitensis* in cattle and small ruminants, respectively (Rubach et al., 2013). Pastoralists commonly co-graze these species together and transmission of *B. abortus* to sheep has been reported where the two species co-graze (McDermott & Arimi, 2002). The same is not usually true for *B. melitensis* which is usually restricted to small ruminants in SSA (Sanogo et al., 2013). However, a recent unpublished study sampling cattle and sheep in central Cameroon detected both *B. abortus* and *B. melitensis* in both species (Mitterran et al., 2020). As around 30% of cattle keepers in this study (both pastoral and dairy) kept small ruminants with their cattle (Kelly et al., 2016), these mixed populations could act as sources of infection for each other, especially if birthing within the same environments.

A review of *C. burnetii* in cattle populations in SSA reported a higher seroprevalence in cattle populations in West and North Africa (18%–55%) compared to the rest of the continent (≤13%) (Vanderburg et al., 2014). We have reported a prevalence at the low end of this estimate and much lower than the previous estimate of 31% (Scolamacchia et al., 2010).

For *C. burnetii*, we found an association with decreased precipitation and denser forested environments. *Coxiella burnetii* is spread through ticks (Hornok et al., 2014; Mtshali et al., 2015; Reye et al., 2012) and can be found in high concentrations in humid forested environments (Silatsa et al., 2019). The organism can also persist for long periods in dry environments. It could be postulated that animals may be more exposed to *C. burnetii* in these environmental conditions. *Leptospira hardjo* seroprevalence was previously reported to be high in cattle populations sampled in Tanzania (30.3%) (Schoonman & Swai, 2010), Kenya (23.5%) (Nthiwa et al., 2019) and in Madagascar (20% detected by qPCR in slaughtered cattle) (Rahelinirina et al., 2019). Seroprevalence of *L. hardjo* in the pastoral cattle was similar to previous estimates in the Adamawa Region of Cameroon (Scolamacchia et al., 2010) and other regions in SSA.

Our study identified transhumance to be protective for exposure to *Leptospira spp* at a herd level, which was also observed in a previous study (Mazeri et al., 2012). *Leptospira hardjo* is usually transmitted through direct or indirect contact with infected urine and through sharing contaminated stagnant water sources. For example, transhumance may decrease contact with urine by sharing of large rapidly moving freshwater bodies, such as streams and rivers (Appendix S1).

Exposure to *C. burnetii and L. hardjo* was common in pastoral cattle. Increasing age was identified as a risk factor for seropositivity to both pathogens, almost certainly due to accumulating opportunity for exposure (Mazeri et al., 2012). Considering the risk factors identified in this study, managing cattle in a manner where they are in close contact with one another in confined spaces could increase their exposure to either of these infections, although this work does not offer any evidence for a direct link to mechanisms for shared risk of co-infection and further work is needed.

In the dairy cattle, exposure to *Leptospira* spp. was much lower than that observed in the pastoral herds (1.7%, CI: 0.0%–4.9%) and we did not detect exposure to *C. burnetii*, suggesting that management of dairy herds reduces their exposure to these pathogens. Smallholder dairy cattle have been managed separately from pastoral cattle as dairy cooperatives for several decades with minimal crossbreeding. They are managed very differently to pastoral cattle with as much as 97.8% of dairy cattle being housed (Kelly et al., 2016). Such zero grazed systems may be protective against exposure to *C. burnetii* through limiting contact with potential tick vectors. In this study, dairy cattle are mainly provided water in troughs, compared to natural water sources for pastoral cattle (Kelly et al., 2016). Lower prevalence for *L. hardjo* in dairy cattle may be associated with the way dairy cattle are given water as transmission is often associated with large bodies of water, such as flooded grazing (Schoonman & Swai, 2010).

Like many SSA countries, Cameroon is yet to undertake coordinated control measures against these zoonoses in livestock populations, unlike in many high-income countries. Although our study samples were collected in 2013, in the absence of national control measures, it is likely that these bacterial zoonoses are still likely circulating within cattle populations in Cameroon. With increasing demand for dairy products (Bayemi et al., 2005) and size of the cattle population (Department & C. N. S, 2012), increased contact between individual cattle through intensification is likely to accelerate risk of transmission. Consequently, keepers of these animals and the consumers of their products are at risk of becoming infected with these zoonotic pathogens. In Cameroon, there are reports that hospital patients presenting with chronic fever (which can be caused by livestock associated bacterial infections) are often misdiagnosed as malaria (Parsel et al., 2017). Despite bacterial zoonoses potentially contributing to differential diagnoses for chronic fever, there remains a disconnect between human and animal studies with very few studies describing the epidemiology of livestock-originated zoonoses in people. In the Adamawa Region of Cameroon, an abattoir-based study demonstrated exposure to *Brucella spp*. in both abattoir workers (5.6%) and pregnant women (0.28%). These people were assumed to have become infected at their work during slaughter of animals (abattoir workers) or through consumption of milk (pregnant women) (Awah-Ndukum, Mouiche, Kouonmo-Ngnoyum, et al., 2018). There is currently no information available about human exposure levels to *C. burnetii* or *L. hardjo* specifically in cattle keeping communities. Consequently, there is a need for multispecies surveillance studies to understand transmission dynamics, having the potential to highlight the significance of transmission between humans, cattle, other livestock and wildlife (Fèvre et al., 2017; Simpson et al., 2018). Such studies could assist developing consistent public health messaging campaigns to warn people of the risks, for example, the importance of safe disposal of placenta when handling animals, their carcasses and the importance of hygienic milk processing practices.

## Supplementary Material

Supporting Information

## Figures and Tables

**Table 1:** Descriptive summary of pastoral and dairy cattle samples

	North west region *n* = 750 (95% CI)	Vina division *n* = 748 (95% CI)	Dairy *n* = 60 (95% CI)
Sex			
Male	29.7% (26.8%–32.7%)	26.5% (22.7%–30.8%)	1.7% (0.0%–4.9%)
Female	70.3% (67.3%–73.2%)	73.5% (69.2%–77.3%)	98.3% (95.1%–100%)
Age (by dentition score [DS])			
Young (<2 years: DS 0)	30.8% (28.5%–33.2%)	30.4% (27.4%–33.6%)	3.3% (0.0%–7.9%)
Adult (≥2 and <5 years: DS 1–4)	66.9% (64.0%–69.7%)	66.8% (63.4%–70.0%)	88.3% (80.1%–96.5%)
Old adult (≥5 years: DS 5)	2.3% (1.2%–4.1%)	2.7% (1.7%–4.2%)	8.3% (1.2%–15.4%)
Breed			
Holstein Friesian	0.0% (0.0%–0.5%)	0.0% (0.0%–0.5%)	98.3% (95.1%–100%)
Gudali	0.0% (0.0%–0.5%)	83.5% (78.1%–88.8%)	0.0% (0.0%–6.0%)
Mixed breed	63.9% (54.6%–72.2%)	14.6% (10.0%–20.7%)	0.0% (0.0%–6.0%)
Fulani	36.1% (27.8%–45.4%)	2.0% (0.9%–4.1%)	0.0% (0.0%–6.0%)
Body condition score (BCS)			
Thin (1–2)	58.1% (53.8%–62.3%)	24.2% (20.8%–28.0%)	25.0% (14.0%–36.0%)
Moderate (3)	37.0% (33.4%–40.8%)	56.4% (53.0%–59.8%)	50.0% (37.2%–62.8%)
Fat (4–5)	4.9% (3.1%–7.5%)	19.3% (15.9%–23.1%)	25.0% (14.0%–36.0%)
Anthelmintic treatment in the past 12 months			
Treated	47.3% (39.2%–55.4%)	30.9% (25.3%–37.1%)	100.0% (94.0%–100%)

**Table 2:** Summary of seroprevalence in of pastoral and dairy cattle samples

	North west region *n* = 750 (95% CI)	Vina division *n* = 748 (95% CI)	Dairy *n* = 60 (95% CI)
*Brucella spp.*	4.2% (2.5%–7.0%)	1.1% (0.5%–2.4%)	5.0% (0.0%–10.6%)
*C. burnetii*	14.6% (11.8%–18.0%)	12.4% (9.6%–15.9%)	0.0% (0.0%–6.0%)
*L. hardjo*	30.7% (26.3%–35.5%)	35.9% (31.3%–40.7%)	1.7%, (0.0%–4.9%)

**Table 3:** Final MLR model factors associated with *Brucella* spp. seropositivity in pastoral cattle (*n* = 1,498)

Model		brucPN ~ SHEEPO + FENCEC + strata1 + (1|HER_ID) *Binary outcome: Brucella spp. seropositive.*		
Variable		Level	OR (95% CI)	*p* value
Keep or rear sheep		No	Ref	<.01
		Yes	3.85 (1.68–8.82)	
Fencing cattle in at night		No	Ref	<.01
		Yes	3.06 (1.33–7.06)	
Study site		North west region	Ref	.03
		Vina Division	0.34 (0.13–0.88)	

*Note:* Outcome variable: *Brucella s*pp. seropositivity (brucPN). Explanatory variables included as fixed effects include Keep or rear sheep (SHEEPO); Fencing cattle in at night (FENCEC); Study site (strata1). Herd identifier (HER_ID) was included as random effect.

**Table 4:** Final MLR model factors associated with *C. burnetii* seropositivity in pastoral cattle (*n* = 1,498)

Model		QfevPN ~ ANIDEN + LeptoPN + tPrecipR + TreeR + strata1 + (1|HER_ID) *Binary outcome: C. burnetii seropositive.*		
Variable		Level	OR (95% CI)	*p* value
Age (by dentition score [DS])		Young (<2 years: DS 0)	Ref	
		Adult (≥ 2 and <5 years: DS 1–4)	3.12 (2.00–4.85)	<.01
		Old adult (≥5 years: DS 5)	2.37 (0.83–6.76)	.11
*L. hardjo* seropositivity		Negative	Ref	
		Positive	1.82 (1.33–2.49)	<.01
Precipitation (mm/month)			0.78 (0.66–0.94)	<.01
Tree density (total area of trees within 5 km)			2.06 (1.18–3.58)	.01
Study site		North west region	Ref	
		Vina division	0.82 (0.58–1.15)	.25

*Note:* Outcome variable: *C. burnetii* seropositivity (QfevPN). Explanatory variables included as fixed effects include Age (ANIDEN); *L. hardjo* seropositivity (LeptoPN); Precipitation (tPrecipR); Tree density (TreeR); Study site (strata1). Herd identifier (HER_ID) was included as random effect.

**Table 5:** Final MLR model factors associated with *Leptospira interrogans serovar hardjo* seropositivity in pastoral cattle (*n* = 1,498)

Model		QfevPN ~ ANIDEN + LeptoPN + TRACAT + strata1 + (1|HER_ID) *Binary outcome: C. burnetii seropositive.*		
Variable		Level	OR (95% CI)	*p* value
Age (by dentition score [DS])		Young (<2 years: DS 0)	Ref	
		Adult (≥2 and <5 years: DS 1–4)	3.53 (2.63–4.73)	<.01
		Old adult (≥5 years: DS 5)	3.40 (1.60–7.24)	<.01
*C. burnetii* seropositivity		Negative	Ref	
		Positive	1.83 (1.32–2.52)	<.01
Undertake transhumance		No	Ref	
		Yes	0.65 (0.44–0.97)	.04
Study site		North west region	Ref	
		Vina division	1.08 (0.77–1.52)	.65

*Note:* Outcome variable: *L. hardjo* seropositivity (LeptoPN). Explanatory variables included as fixed effects include Age (ANIDEN); *C. burnetii* seropositivity (QfevPN); Undertake transhumance (TRACAT); Study site (strata1). Herd identifier (HER_ID) was included as random effect.

## Impacts

- Exposure to important production diseases and occupational zoonoses are still widespread in Cameroonian cattle, including *Brucella* species, *Coxiella burnetii* and *Leptospira hardjo*. Their presence poses a risk to the health and livelihoods of animal-keeping communities in Cameroon.
- This is the first time that *Brucella* species and *Leptospira hardjo* were detected in dairy cattle in Cameroon, highlighting the potential zoonotic risk to the wider milk-consuming human population.
- For pastoral cattle, seroprevalence varied by pathogen and locality. Risk factors for exposure were mainly associated with local husbandry practices and co-infections. Understanding the collective risk factors highlights an opportunity for coordinated control.
