# Supplementary material for: The epidemiology of bacterial zoonoses in pastoral and dairy cattle in Cameroon, Central Africa
Source: Zoonoses Public Health. Author manuscript; Available in PMC 2024 Dec 22. (PMC7617252; doi:10.1111/zph.12865)
Supplement: Supporting Information [file EMS201899-supplement-Supporting_Information.docx]

**Appendix S1.** A descriptive summary of cattle keeper information in pastoral and dairy cattle from cross-sectional studies.

|  | North West Region  Pastoralists  (95% CI, n=50) | | | | Vina Division  Pastoralists  (95% CI, n=50) | | | North West Region  Dairy Farmers  (95% CI, n=46) | |
| --- | --- | --- | --- | --- | --- | --- | --- | --- | --- |
| PARTICIPANTS | | | | | | | | | |
| Gender | | | | | | | | | |
| *Male* | **97.8%** (86.4-99.7%) | | | | **100%** (92.9-100%) | | | **56.5%** (42.0-71.0%) | |
| *Female* | **2.2%** (0.0-11.6%) | | | | **0.0%** (0.0-7.1%) | | | **43.5%** (29.0-58.0%) | |
| Age | | | | | | | | | |
| Mean (Years) | **41.0** (37.0-44.9) | | | | **39.2** (35.3-43.4) | | | **45.8** (42.4-49.3) | |
| Schooling level | | | | | | | | | |
| *None* | **63.2%** (50.0-74.7%) | | | | **74.0%** (60.6-84.2%) | | | **0%** (0.0-7.7%) | |
| *Primary* | **24.8%** (14.9-38.4%) | | | | **23.9%** (14.1-37.4%) | | | **76.1%** (63.3-88.5%) | |
| *Secondary* | **8.1%** (3.1-19.2%) | | | | **2.0%** (0.2-12.3%) | | | **10.9%** (1.8-20.0%) | |
| *Higher* | **3.9%** (0.1-13.0%) | | | | **0.0%** (0.0-7.1%)) | | | **5.5%** (4.0-6.9%) | |
| Mean time kept cattle (Years) | **26.5** (22.4-30.5) | | | | **17.7** (13.7-21.4) | | | **5.5** (4.0-6.9) | |
| Ethnic group | | | | | | | | | |
| *Mbororo* | **89.4%** (77.4-95.4%) | | | | **17.6%** (9.9-29.5%) | | | **0.0%** (0.0-7.7%)) | |
| *Fulbe* | **2.0%** (0.2-12.7%) | | | | **66.1%** (53.0-77.1%) | | | **0.0%** (0.0-7.7%)) | |
| *Other** | **8.6%** (3.3-20.2%) | | | | **16.2%** (8.3-29.2%) | | | **100%** (92.3-100%) | |
| Cattle keeper type | | | | | | | | | |
| *Caretaker* | **20.2%** (11.9-32.4%) | | | | **27.5%** (17.2-40.9%) | | | **6.5%** (0.0-13.7%) | |
| *Herdsman* | **31.9%** (20.3-46.2%) | | | | **45.5%** (34.5-60.8%) | | | **0.0%** (0.0-7.7%) | |
| *Owner* | **47.9%** (35.3-60.8%) | | | | **27.5%** (17.2-40.9%) | | | **93.5%** (86.3-100%) | |
| Length of time kept cattle | | | | | | | | | |
| *Mean (Years)* | **26.5** (22.4-30.5) | | | | **17.7** (13.7-21.4) | | | **5.5** (4.0-6.9) | |
| HERD | | | | | | | | | |
| Size | | | | | | | | | |
| *Mean* | **50** (45-55) | | | | **38** (34-43) | | | **3** (2-3) | |
| Breeds kept | | | | | | | | | |
| *Mixed* | **63.9%** (54.6-72.2%) | | | | **15.0%** (9.7-30.1%) | | | **0.0%** (0.0-7.7%) | |
| *Red Fulani* | **16.1%** (10.1-24.7%) | | | | **0.0%** (0.0-7.1%) | | | **0.0%** (0.0-7.7%) | |
| *White Fulani* | **20.0%**  (12.9-29.8%) | | | | **1.5%** (0.2-1.0%) | | | **0.0%** (0.0-7.7%) | |
| *Gudali* | **0.0%** (0.0-7.1%) | | | | **83.5%** (78.1-88.8%) | | | **0.0%** (0.0-7.7%) | |
| *Holstein Friesian* | **0.0%** (0.0-7.1%) | | | | **0.0%** (0.0-7.1%) | | | **100%** (92.3-100%) | |
| OTHER SPECIES | | | | | | | | | |
| What other animals, other than cattle, do you currently keep or rear at your homested? | | | | | | | | | |
| *Sheep* | | **45.2%** (33.7-57.1%) | | **28.8%** (18.7-41.6%) | | | **23.9%** (11.5-36.4%) | | |
| *Goats* | | **29.9%** (19.2-43.4%) | | **27.2% (**16.7-41.0%) | | | **30.4%** (20.9-48.7%) | | |
| *Poultry* | | **75.4%** (61.2-85.7%) | | **65.4%** (51.1-77.4%) | | | **63.0%** (48.9-77.1%) | | |
| *Cats* | | **42.9%** (30.7-55.9%) | | **43.4%** (31.2-56.6%) | | | **26.0%** (13.3-38.9%) | | |
| *Dogs* | | **36.4%** (24.5-50.5%) | | **31.3%** (20.5-44.5%) | | | **54.3%** (42.0-71.0%) | | |
| *Horses^* | | **39.9%** (28.2-53.0%) | | **2.0%** (0.2-12.4%) | | | **2.2%** (0.0-6.4%) | | |
| Do any of these cattle presented come in contact with the following wild animals whilst grazing? | | | | | | | | | |
| *Buffalo* | | **0.0%** (0.0-7.1%) | | **0.0%** (0.0-7.1%) | | | **NA** | | |
| *Antelope* | | **49.8%** (36.0-63.6%) | | **76.4%** (65.4-84.7%) | | | **NA** | | |
| *Warthog* | | **11.9%** (5.4-24.0%) | | **38.2%** (27.2-50.6%) | | | **NA** | | |
| If you cattle go on transhumance; do any of these cattle presented come in contact with the following wild animals whilst on transhumance? | | | | | | | | | |
|  | | (n=22) | | (n=3) | | | NA | | |
| *Buffalo* | | **27.3%** (12.9-48.8%) | | **0.0%** (0.0-70.8%) | | | **NA** | | |
| *Antelope* | | **81.8%** (60.6-92.9%) | | **66.7%** (16.3-95.3%) | | | **NA** | | |
| *Warthog* | | **31.8%** (16.1-53.2%) | | **66.7%** (16.3-95.3%) | | | **NA** | | |
| CATTLE INFECTIOUS DISEASE CONTROL | | | | | | | | | |
| Have the cattle presented been vaccinated? | | | | | | | | | |
| *Yes* | | | **98.0%** (87.6-99.7%) | | | **100%** (92.9-100%) | | | **97.8%** (93.6-100%) |
| Have you treated the cattle presented with an anthelmintic? | | | | | | | | | |
| *Yes* | | | **93.9%** (82.6-98.1%) | | | **84.2%** (71.2-92.0%) | | | **100%** (92.3-100%) |
| Have you treated any of the cattle presented for trypanosomiasis? | | | | | | | | | |
| *Yes* | | | **41.9%** (29.1-56.0%) | | | **77.7%** (65.9-86.2%) | | | **0.0%** (0.0-8.6%) |
| CATTLE REPRODUCTION PRACTICES | | | | | | | | | |
| Have you used natural breeding? | | | | | | | | | |
| *Yes* | | | **100%** (92.9-100%) | | | **100%** (92.9-100%) | | | **89.1%** (80.0-98.2%) |
| If so; What breed of bull do you use for natural breeding? | | | (n=50) | | | (n=50) | | | (n=41) |
| *Fulani* | | | **39.6%** (26.7-52.5%) | | | **0.0%** (0.0-7.1%) | | | **0.0%** (0.0-8.6%) |
| *Gudali* | | | **0.0%** (0.0-7.1%) | | | **91.4%** (85.5-97.2%) | | | **0.0%** (0.0-8.6%) |
| *Mixed Breed* | | | **62.5%** (50.3-74.7%) | | | **5.4%** (0.0-11.3%) | | | **0.0%** (0.0-8.6%) |
| *Holstein-Friesian* | | | **0.0%** (0.0-7.1%) | | | **0.0%** (0.0-7.1%) | | | **100%** (91.4-100%) |
| Have you used artificial insemination (AI)? | | |  | | |  | | |  |
| *Yes* | | | **10.2%** (4.4-22.01%) | | | **2.0%** (0.0-5.8%) | | | **8.0%** (1.7-14.4%) |
| If so; What breed of bull do you use for AI? | | | (n=5) | | | (n=1) | | | (n=5) |
| *European Breed*  *(Bos taurus)* | | | **100%** (47.8-100%) | | | **100%** (2.5-100%) | | | **100%** (47.8-100%) |
| CATTLE GRAZING AND HOUSING PRACTICES | | | | | | | | | |
| Have the presented cattle been kept housed the majority of time? | | | | | | | | | |
| *Yes* | | | **0.0%** (0.0-7.1%) | | | **0.0%** (0.0-7.1%) | | | **97.8%** (93.6-100%) |
| Have these cattle grazed open pasture? | | | | | | | | | |
| *Yes* | | | **100%** (92.9-100%) | | | **100%** (92.9-100%) | | | **4.35%** (0.0-10.3%) |
| Do you keep these cattle presented in a fenced enclosure overnight? | | | | | | | | | |
| *Yes* | | | **54.7%** (43.1-66.3%) | | | **17.1%** (8.8-25.3%) | | | **100%** (15.8-100%) |
| If so; What type of pasture do these cattle graze? | | | (n=50) | | | (n=50) | | | (n=2) |
| *Natural pasture* | | | **98.7%** (96.1-100%) | | | **100%** (92.9-100%) | | | **0.0%** (0.0-84.2%) |
| *Improved pasture* | | | **1.3%** (0.0-3.9%) | | | **0.0%** (0.0-7.1%) | | | **100%** (15.8-100%) |
| Where do the presented cattle drink from on a regular basis? | | | | | | | | | |
| *Streams* | | | **97.9%** (86.6-99.7%) | | | **97.9%** (86.6-99.7%) | | | **0.0%** (0.0-8.6%) |
| *Water troughs* | | | **0.0%** (0.0-7.1%) | | | **0.0%** (0.0-7.1%) | | | **100%** (92.3-100%) |
| *Water canals* | | | **1.9%** (0.3-12.3%) | | | **0.0%** (0.0-7.1%) | | | **0.0%** (0.0-8.6%) |
| *Lakes and ponds* | | | **1.9%** (0.3-12.3%) | | | **0.0%** (0.0-7.1%) | | | **0.0%** (0.0-8.6%) |
| Did any of these cattle presented go on transhumance? | | | | | | | | | |
| *Yes* | | | **43.8%** (31.4-57.1%) | | | **6.2%** (2.0-17.7%) | | | **0.0%** (0.0-8.6%) |
| CATTLE TRADE PRACTICES | | | | | | | | | |
| Did you in the past 12 months: | | | | | | | | | |
| *Purchase cattle?* | | | **41.8%** (30.0-54.7%) | | | **49.7%** (36.4-62.9%) | | | **8.7%** (0.4-16.9%) |
| *Sell cattle?* | | | **93.8%** (83.2-97.9%) | | | **83.9%** (71.3-91.6%) | | | **37.0%** (22.9-51.1%) |
| If purchased or sold cattle did you do so at markets? | | | (n=42) | | | (n=44) | | | (n=17) |
| *Yes* | | | **83.4%** (70.0-91.5%) | | | **87.8%** (75.4-94.4%) | | | **11.8%** (0.0-27.6%) |

***^*^****Non-Fulani/ Grassland ethnic group.*

^^^*Response not recorded for 1 participant (n=49).*

**Appendix S2.** Univariate analysis for inclusion risk factor multivariable logistic regression models in pastoral cattle (n=1498) for *Brucella* spp, *C. burnetii* and *Leptospira hardjo* spp. seropositivity. Herd level factors apply to the previous 12 months. Data on cattle density were extracted from the FAO Gridded livestock Atlas v3. These were processed as the total number of livestock within 10km of the animal location. Data on weather and climate from CRU <https://sites.uea.ac.uk/cru/> between 2011 and 2014. Data on landcover form ESA landcover classification <http://www.esa-landcover-cci.org/?q=node/164> . These were processed as the total area of trees, shrubland and grassland within 5km of each sampling site. Prior analysis, ecological variables (x) were centered to start at zero using the following equation $\surd$(x) - min($\surd$ (x)) except landcover variables, which were centered using log(x) - min(log(x)). *=Explanatory variables to included in final MLR model selection (p value <0.2).

| **Variable** | **Code** | **P value** | | |
| --- | --- | --- | --- | --- |
|  |  | ***Brucella spp.* (brucPN)** | ***C. burnetii* (QfevPN)** | ***L. hardjo***  **(LeptoPN)** |
| Breed | ABREED | 0.40 | 0.69 | 0.57 |
| Body condition score | ANIBCS | 0.50 | 0.51 | 0.32 |
| Dentition score | ANIDEN | 0.31 | <0.01* | <0.01* |
| Provided worming treatment in the previous 12 months | QUESWM | 0.90 | <0.01* | <0.01* |
| Number of cattle presented | NUMCTP | 0.82 | 0.69 | 0.88 |
| Keep or rear sheep | SHEEPO | <0.01* | 0.53 | 0.65 |
| Keep or rear goats | GOATSO | 0.31 | 0.28 | 0.44 |
| Keep or rear other livestock | OTHERO | 0.92 | 0.42 | 0.33 |
| Cattle treated with a trypanocide | TRYPRV | 0.14* | 0.46 | 0.41 |
| Artifical breeding conducted | AISBRD | 0.03* | 0.41 | 0.79 |
| Predominant grazing type | GRZTYP | 0.59 | 0.64 | 0.39 |
| Grazing flooded pasture | GRZFLD | 0.39 | 0.30 | 0.82 |
| Number of herds graze pasture with | GRZNHD | 0.66 | 0.68 | 0.55 |
| Co-graze pasture with buffalo | GRZBUF | 0.15* | 0.95 | 0.77 |
| Co-graze pasture with antelope | GRZANT | 0.30 | 0.79 | 0.58 |
| Co-graze pasture with warthogs | GRZHOG | 0.51 | 0.58 | 0.61 |
| House cattle the majority of the time | HOUSEC | 0.94 | 0.85 | 0.59 |
| Fence cattle in at night | FENCEC | <0.01* | 0.10* | <0.01* |
| Drink water from troughs | DRKWTT | 0.61 | 0.60 | 0.33 |
| Drink water from canals | DRKWTC | 0.99 | 0.90 | 0.87 |
| Drink water from streams | DRKSTR | 0.84 | 0.67 | 0.78 |
| Drink water from lakes or ponds | DRKLOP | 0.65 | 0.56 | 0.95 |
| Drink with other herds | DRKCON | 0.60 | 0.61 | 0.53 |
| Undertake transhumance | TRACAT | 0.12* | 0.83 | 0.04* |
| Buy cattle at markets | BUYCAT | 0.36 | 0.43 | 0.62 |
| *Brucella spp.* seropositive | brucPN | NA | 0.26 | 0.25 |
| *C. burnetii* seropositive | QfevPN | 0.25 | NA | <0.01* |
| *L. hardjo* spp. seropositive | LeptoPN | 0.29 | 0.01* | NA |
| Local cattle density | cattleR | 0.57 | 0.02* | 0.91 |
| Elevation (m) | elevRescale | 0.96 | 0.03* | 0.58 |
| Precipitation (mm/month) | tPrecipR | 0.79 | <0.01* | 0.75 |
| Minimum temperature (^o^C) | tMinR | 0.73 | 0.25 | 0.87 |
| Mean temperature (^o^C) | tMeanR | 0.69 | 0.21 | 0.96 |
| Maximum temperature (^o^C) | tMaxR | 0.67 | 0.05 | 0.96 |
| Temperature range (^o^C) | dtrR | 0.51 | 0.80 | 0.96 |
| Water vapour (hPa) | tVapR | 0.74 | <0.01* | 0.55 |
| Tree density | TreeR | 0.08* | 0.08* | 0.48 |
| Shrub density | ShrubR | 0.09* | 0.64 | 0.53 |
| Grass type | GrassR | 0.19* | 0.93 | 0.14* |

**Appendix S3.** Backwards stepwise selection of final risk factor multivariable logistic regression models in pastoral cattle (n=1498) for a. *Brucella* spp. (brucPN) b. *C. burnetii*  (QfevPN) and c. *Leptospira hardjo* spp. (LeptoPN) seropositivity. Study site (strata1) were included in all models as a fixed effect and herd (HER_ID) as a random effect. Other abbreviations for each explanatory variable included in each model are included in supplementary material 2 and * indicates final model selected.

1. *Brucella* spp.

| **Model** | **AIC** |
| --- | --- |
| brucPN ~ SHEEPO + TRYPRV + AISBRD + GRZBUF + FENCEC + TRACAT + TreeR + ShrubR + GrassR + strata1 +(1\|HER_ID) | 327.58 |
| brucPN ~ SHEEPO + TRYPRV + AISBRD + GRZBUF + FENCEC + TRACAT + TreeR + ShrubR + strata1 +(1\|HER_ID) | 326.53 |
| brucPN ~ SHEEPO + TRYPRV + AISBRD + GRZBUF + FENCEC + TRACAT + ShrubR + strata1 +(1\|HER_ID) | 325.63 |
| brucPN ~ SHEEPO + TRYPRV + GRZBUF + FENCEC + TRACAT + ShrubR + strata1 +(1\|HER_ID) | 323.71 |
| brucPN ~ SHEEPO + TRYPRV + FENCEC + TRACAT + ShrubR + strata1 +(1\|HER_ID) | 322.43 |
| brucPN ~ SHEEPO + TRYPRV + FENCEC + TRACAT + strata1 +(1\|HER_ID) | 321.39 |
| brucPN ~ SHEEPO + FENCEC + TRACAT + strata1 +(1\|HER_ID) | 320.60 |
| brucPN ~ SHEEPO + FENCEC + strata1 +(1\|HER_ID)* | 319.70 |

*ICC= 0.14

1. *Coxiella burnetii*.

| **Model** | **AIC** |
| --- | --- |
| QfevPN ~ ANIDEN + QUESWM + FENCEC + LeptoPN + cattleR + tPrecipR + tMaxR + TreeR + strata1 +(1\|HER_ID) | 1146.8 |
| QfevPN ~ ANIDEN + QUESWM + FENCEC + LeptoPN + cattleR + tPrecipR + tMaxR + TreeR + strata1 +(1\|HER_ID) | 1144.8 |
| QfevPN ~ ANIDEN + QUESWM + LeptoPN + cattleR + tPrecipR + tMaxR + TreeR + strata1 +(1\|HER_ID) | 1143.0 |
| QfevPN ~ ANIDEN + QUESWM + LeptoPN + cattleR + tPrecipR + TreeR + strata1 +(1\|HER_ID) | 1142.0 |
| QfevPN ~ ANIDEN + LeptoPN + cattleR + tPrecipR + TreeR + strata1 +(1\|HER_ID) | 1142.0 |
| QfevPN ~ ANIDEN + LeptoPN + tPrecipR + TreeR + strata1 +(1\|HER_ID)* | 1141.7 |

*ICC= 0.02

1. *L. hardjo* spp.

| **Model** | **AIC** |
| --- | --- |
| LeptoPN ~ ANIDEN + QUESWM + FENCEC + TRACAT + QfevPN + GrassR + strata1 +(1\|HER_ID) | 1785.6 |
| LeptoPN ~ ANIDEN + FENCEC + TRACAT + QfevPN + GrassR + strata1 +(1\|HER_ID) | 1783.6 |
| LeptoPN ~ ANIDEN + FENCEC + TRACAT + QfevPN + strata1 +(1\|HER_ID) | 1782.5 |
| LeptoPN ~ANIDEN + QfevPN + TRACAT + strata1 +(1\|HER_ID)* | 1782.1 |

*ICC= 0.08
